# Conservative Management of Focal Chondral Lesions of the Knee and Ankle: Current Concepts

**DOI:** 10.3390/cells14231899

**Published:** 2025-12-01

**Authors:** Filippo Migliorini, Raju Vaishya, Julian Koettnitz, Madhan Jeyaraman, Luise Schäfer, Jörg Eschweiler, Francesco Simeone

**Affiliations:** 1 Department of Trauma and Reconstructive Surgery, University Hospital of Halle, Martin-Luther University Halle-Wittenberg, 06097 Halle (Saale), Germany; 2 Department of Orthopaedic and Trauma Surgery, Academic Hospital of Bolzano (SABES-ASDAA), Via Lorenz Böhler 5, 39100 Bolzano, Italy; 3 Department of Life Sciences, Health, and Health Professions, Link Campus University, Via del Casale di San Pio V, 00165 Rome, Italy; 4 Department of Orthopaedic and Trauma Surgery, Eifelklinik St.Brigida, Kammerbruchstr. 8, 52152 Simmerath, Germany; 5 Department of Orthopaedics and Joint Replacement Surgery, Indraprastha Apollo Hospital, Sarita Vihar, New Delhi 110076, India; 6 Department of Orthopaedic and Trauma Surgery, University Hospital of RUB-Bochum, 32545 Bad Oeynhausen, Germany; 7 Department of Orthopaedics, ACS Medical College and Hospital, Dr MGR Educational and Research Institute, Chennai 600077, Tamil Nadu, India; 8 Department of Regenerative Medicine, Agathisha Institute of Stemcell and Regenerative Medicine (AISRM), Chennai 600030, Tamil Nadu, India; 9 Department of Trauma and Reconstructive Surgery, BG Klinikum Bergmannstrost Halle, 06112 Halle (Saale), Germany

**Keywords:** joint preservation, focal cartilage injury, non-operative treatment, rehabilitation, platelet-rich plasma, hyaluronic acid, ankle cartilage defect

## Abstract

Focal chondral defects of the knee and ankle remain a challenging clinical condition, particularly in young and active patients, as they often cause pain, mechanical symptoms, and functional limitation without necessarily progressing to osteoarthritis (OA). This narrative review summarises current evidence on non-operative strategies for managing focal chondral lesions in non-arthritic joints, emphasising the role of rehabilitation as the central component of care. A thematic literature search was conducted across major databases for studies published between 2000 and 2025, selecting articles based on clinical relevance. Structured rehabilitation programmes based on load optimisation, neuromuscular retraining, and progressive strengthening represent the foundation of conservative management. Pharmacological agents and intra-articular injectables may provide temporary relief, although the evidence supporting their efficacy remains heterogeneous and primarily short-term. Nutraceuticals and physical modalities show encouraging but inconsistent results, limited by methodological variability and undefined dosing. Overall, conservative treatment should be tailored to the individual patient’s biomechanical and biological profile, integrating rehabilitation with selected adjuncts when appropriate. Future research should focus on developing standardised rehabilitation protocols, identifying predictors of recovery, and clarifying the biological mechanisms that sustain symptom improvement in focal cartilage pathology.

## 1. Introduction

Focal chondral defects of the knee and ankle represent a substantial and often underappreciated clinical problem, particularly in active and younger populations. Articular cartilage injuries are observed in 60–66% of knees undergoing arthroscopy, with a median patient age ranging from 30 to 39 years [1,2]. Focal full-thickness chondral defects have a prevalence ranging from 4.2 to 6.2% among all patients undergoing knee arthroscopy, and they may affect up to 36% of athletes [3]. These lesions carry significant morbidity owing to pain, functional limitation, early joint degeneration and increased risk of subsequent osteoarthritis (OA), thereby imposing a meaningful burden on patients, health care systems and society at large.

Focal chondral defects impair joint function and quality of life through pain, swelling, and load-related mechanical symptoms [4,5,6,7]. Yet, a substantial proportion of patients can be managed without surgery when treatment is aligned with cartilage biology and joint biomechanics [8]. Non-operative care aims to relieve pain, improve function, and protect the joint microenvironment, while realistic expectations are set regarding the limited capacity of adult articular cartilage for structural regeneration [9,10]. The target population in this review comprises symptomatic focal defects in non-arthritic knees and ankles, where joint preservation is the priority and surgery can be deferred or avoided in selected cases. This scope is deliberately separated from OA because pain generators, inflammatory tone, structural progression, and responsiveness to therapies differ in a clinically meaningful way [11,12]. Adult hyaline cartilage is avascular, alymphatic and sparsely populated by chondrocytes, and its mechanical competence emerges from an organised type II collagen network interwoven with proteoglycans that create a high fixed charge density and low permeability [13,14,15,16]. After injury, endogenous repair tends to generate fibrocartilage with inferior composition and mechanics, which explains why a restitutio ad integrum is uncommon and why non-surgical strategies primarily aim to modulate synovitis, optimise load distribution and enhance neuromuscular control rather than to recapitulate native histology [17,18,19]. Structure–function work has shown that alterations in collagen content and architecture influence compressive properties at least as much as glycosaminoglycan content in repair tissues, reinforcing the idea that biological or mechanical interventions that fail to restore an ordered collagen network will yield limited structural benefit even if symptoms improve [20,21]. Cellular fate in the osteochondral unit is further shaped by oxygen tension, angiogenic cues and substrate stiffness, with computational and experimental studies indicating that mesenchymal progenitors are steered by hypoxia and mechanical context and that excessive vascular ingress in the chondral zone promotes hypertrophy. In contrast, inadequate perfusion impairs matrix synthesis, insights that inform expectations for conservative therapies and contextualise why clinical benefit does not always mirror durable structural change [22]. It should also be recognised that structural restoration of the cartilage surface is uncommon, and that the main benefits of conservative treatment are clinical and functional rather than morphological [9,23]. Within this complex biological framework, the pillars of conservative care are structured rehabilitation, education, and load management, complemented, where appropriate, by intra-articular injectables and the cautious use of oral agents [8,9,24]. Rehabilitation programmes prioritise progressive closed-chain strengthening, proximal hip and trunk control, neuromuscular training, and gait retraining, with bracing and taping used as symptom accelerators in selected phenotypes with maltracking or instability [25,26]. Intra-articular options include hyaluronic acid and platelet-rich plasma, with the latter being increasingly preferred in carefully selected non-arthritic phenotypes. Corticosteroids are reserved as a short-term rescue for florid synovitis rather than as a disease-modifying tool [27,28,29]. Cell-based injectables and bone marrow aspirate concentrates sit largely within surgical workflows, and their purely non-operative use in focal lesions remains investigational [30,31]. Oral and systemic agents, such as non-steroidal anti-inflammatory drugs, can help with pain control. In contrast, targeted modulation of prostaglandin or prostacyclin signalling and monoclonal antibodies are not yet supported by human trials for focal defects [8,32,33]. Hyperbaric oxygen therapy has biological plausibility but lacks robust clinical validation in this setting; therefore, nutraceuticals should be framed around correcting documented deficiencies rather than promising regeneration [34,35]. Finally, the knee and the ankle present distinct loading environments and contact mechanics that shape both symptoms and response to therapy, with the patellofemoral compartment adding substantial shear in deeper flexion and the talar dome operating under high congruent compression, differences that will be addressed explicitly when indications and practical guidance are discussed for each joint [36,37].

This narrative review discusses current evidence on non-operative strategies for symptomatic focal chondral defects in non-arthritic knee and ankle joints. In the present review, conservative management refers exclusively to non-surgical, non-cell-based strategies, including rehabilitation, physical modalities, pharmacotherapy, and intra-articular injectables, while advanced biologic or surgical options were deliberately excluded.

## 2. Methods

All the clinical studies investigating the conservative management of chondral defects of the knee and the ankle were accessed. Only studies published in peer-reviewed journals were considered. Given the author’s language capabilities, articles in English, German, Italian, French, and Spanish were eligible. Studies with levels I to IV of evidence, as defined by the Oxford Centre for Evidence-Based Medicine [38], were considered. In October 2025, the following databases were accessed: PubMed, Web of Science, and Scopus. The Medical Subject Headings (MeSH) used for the database search are reported in Table 1.

## 3. Biology of Cartilage Injury and Repair

Adult articular cartilage is avascular and sparsely cellular, and mechanical competence arises from an organised type II collagen network restraining a proteoglycan-rich gel with a high fixed charge density and low permeability. Once the surface is breached, intrinsic repair is limited and fibrocartilage predominates with inferior architecture and mechanics, with structure–function work showing that compressive behaviour in repaired tissue tracks collagen content and organisation at least as closely as glycosaminoglycans; hence, symptomatic improvement may not restore native stiffness or permeability and a full restitutio ad integrum is uncommon [20,39,40,41,42,43]. Chondrocytes sit at the crossroads of anabolism and catabolism, interleukin one and tumour necrosis factor upregulate cyclooxygenase and MMP13, while prostaglandin E2 signalling is context-dependent. Selective EP2 activation reduced early degeneration and suppressed MMP13 in vivo, and, when delivered by a local microsphere depot, enhanced type II collagen positive repair without measurable synovitis, yet effects waned once delivery ceased, underscoring that biological modulation needs sustained local exposure and a permissive mechanical microenvironment to matter clinically [44,45]. The osteochondral unit behaves as a coupled system, where subchondral perfusion, marrow access and endplate stiffness influence cartilage metabolism. Violation of the plate recruits marrow elements and stabilises symptoms but typically yields fibrous or fibrocartilaginous repair with reduced durability; conversely, sclerosis or bone marrow oedema alter load transfer across the tidemark and perpetuate synovial irritation, which justifies conservative strategies that calm synovitis and optimise load instead of chasing histological perfection [46,47]. Oxygen tension is a master regulator; a mechanobiological model predicted that local hypoxia and substrate stiffness steer progenitors and that angiogenesis confined to bone accelerates early matrix while unchecked vessels in cartilage drive hypertrophy and plate advancement, preserving a hypoxic chondral niche, which is therefore desirable [48,49,50]. Human stem cell data concur: continuous hypoxia programmes mesenchymal stromal cells toward an articular phenotype, with upregulation of gremlin-1, frizzled-related protein, and dickkopf-1 and suppression of collagen-10 and MMP-13. In contrast, normoxia favours hypertrophic, calcifying progression after implantation, refining expectations for injectables and supporting rehabilitation that reduces synovitis and abnormal shear while biology is nudged conservatively [51,52].

## 4. Rehabilitation and Load Management

**Figure 1:**
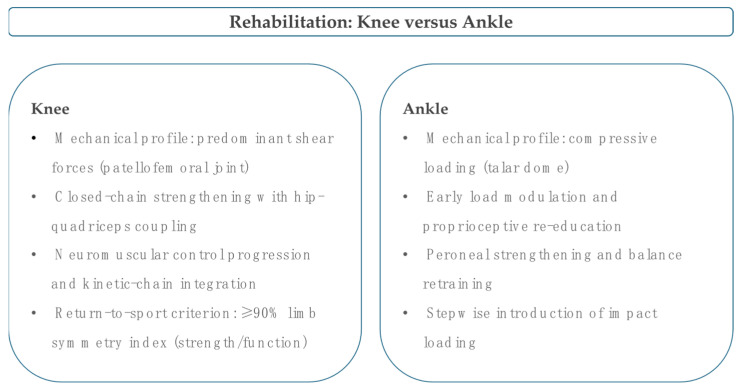
Rehabilitation principles for focal chondral lesions of the knee and ankle.

Rehabilitation is the foundation of non-operative care because symptoms in focal chondral lesions reflect an interplay between nociceptive drivers, synovial irritation, and abnormal load distribution. Programmes must therefore combine education, progressive strengthening, neuromuscular control, and careful exposure to impact [53,54]. In patellofemoral chondromalacia, a randomised trial showed that three weeks of semi-squat work produced larger gains in quadriceps strength, greater reduction in crepitation, and meaningful symptom relief compared with straight leg raises, establishing a preference for closed-chain loading when pain allows and suggesting that short, frequent sessions with strict control of mechanics can accelerate early recovery [55]. The broader question of whether adding arthroscopy to exercise improves anterior knee pain was addressed in a pragmatic trial in which arthroscopy plus an eight-week home programme was no better than the same programme alone at nine months, reinforcing a conservative first approach and justifying escalation only when a structured plan fails and mechanical symptoms persist with supportive imaging [56,57,58]. Bracing can help as a short-term symptom booster in phenotypes with maltracking or irritability during stair descent; a prospective, randomised study found that a medially directed realignment brace added to supervised physiotherapy improved pain and function at six and twelve weeks compared with exercise alone, though effects attenuated over time, so braces should be used as time-limited adjuncts while strength and movement quality are developed, not as substitutes for training [59]. Motion-restricting stabilising braces warrant caution because randomised data after the first patellar dislocation showed increased quadriceps atrophy and a reduced range of motion at early follow-up, without clear prevention of redislocation, compared with a neoprene brace. This indicates that unnecessary restriction should be avoided in chondral phenotypes where muscle preservation and motion are central goals [60,61,62,63]. Exercise content should emphasise progressive closed-chain quadriceps work, hip abductors and external rotators, trunk control, and gait retraining because proximal mechanics influence patellofemoral load and tibial rotation. Adjuncts such as taping can be used to reduce immediate irritability and facilitate quality repetitions, and return to running should be staged based on symptom response and landing mechanics rather than time alone [64,65]. In the ankle, the talocrural joint’s congruent architecture concentrates compressive forces on the talar dome, so early care prioritises graded deloading with protected range, quick transition to proprioceptive and peroneal retraining, and progressive single-leg tasks that restore inversion control and midfoot stiffness during stance. Impact is reintroduced once hopping tests are pain-free and symmetrical, with an acceptable time to stabilise. Persistent mechanical pain or repeated effusions after a well-conducted programme should prompt re-evaluation for bony oedema or unrecognised instability [17,66]. When rehabilitation alone does not provide sufficient relief in carefully selected non-arthritic phenotypes, injections are considered as adjuncts, not replacements for training, and their effects must be interpreted within the mechanical plan, since any symptomatic window gained by an injection should be used to progress load and skill rather than prolong rest [12,27,67]. In this framework, indications are pragmatic; the knee programme is suitable for anterior knee pain with imaging evidence of focal chondral change when pain increases with stairs, prolonged sitting, or deep flexion. It prioritises closed-chain dosing, proximal strengthening, and movement retraining, and a medially directed brace can be trialled for six to twelve weeks to facilitate exposure when maltracking features are evident. The ankle programme suits talar dome lesions presenting with deep ache during stance and push-off, where short deloading quickly gives way to neuromuscular and balance work, and running is delayed until single-leg hops and directional changes are pain-free [53,54,66]. Across both joints, the threshold to persist with conservative care is a meaningful improvement in pain and function within six to twelve weeks, measured with validated scores and specific performance tasks. Failure to progress despite adherence and technically sound dosing should prompt shared decision-making about biologic adjuncts or, if mechanical symptoms dominate, surgical options. However, the default pathway remains rehabilitation-led because it addresses the drivers that injections cannot correct alone [23,68,69,70]. Building on these principles, rehabilitation remains the cornerstone of conservative management for focal chondral lesions. Controlled loading, neuromuscular retraining, and progressive strengthening are central to restoring joint function [71]. Closed-chain exercises are typically performed two to three times per week at moderate intensity, progressing towards endurance and multiplanar loading as tolerated [72]. Return to running and change-of-direction should follow criterion-based progression, requiring a pain-free range of motion, quadriceps strength symmetry above 90%, and stable single-leg control [73]. Common pitfalls include the unnecessary use of motion-restricting braces, which can induce early quadriceps atrophy and limit range of motion without proven benefit [73]. Similarly, excessive non-weight-bearing work may delay recovery, while premature plyometrics can exacerbate symptoms and joint effusion [73,74]. The key practical differences between knee and ankle rehabilitation are summarised in Figure 1.

Patellofemoral shear forces dominate the knee profile and therefore prioritise closed-chain quadriceps-hip strengthening, progression of neuromuscular control, and kinetic-chain integration, with return to sport guided by a limb symmetry index ≥90% for strength and function. The ankle profile is characterised by predominantly compressive loading across the talar dome, requiring early load modulation, proprioceptive re-education, peroneal strengthening and a stepwise introduction of impact loading.

## 5. Pharmacotherapy

Drug therapy for focal chondral lesions should be considered a supportive measure rather than a disease-modifying approach [75,76]. Its primary goal is to control pain and inflammation sufficiently to allow patients to participate fully in rehabilitation. Short courses of simple analgesics or non-steroidal anti-inflammatory drugs can be useful, especially during periods of increased synovitis, provided that potential gastrointestinal, renal, and cardiovascular risks are taken into account and the treatment is kept as brief as possible [75,76,77]. None of the available oral medications has shown convincing evidence of structural cartilage regeneration, so pharmacological treatment must always be temporary and clearly integrated into a wider rehabilitation plan [75,76,77]. Experimental studies have highlighted the biological relevance of prostaglandin signalling in cartilage homeostasis. Selective EP2 receptor activation, for instance, has been shown to limit early degeneration and suppress MMP13 expression. At the same time, local depot delivery can promote type II collagen synthesis, though these benefits tend to wane upon exposure cessation [44,45]. Despite these promising findings, systemic modulation of prostaglandins or prostacyclins remains experimental, and no controlled human trial has yet demonstrated clinical benefit [44,78]. Similarly, monoclonal antibodies targeting inflammatory mediators, systemic corticosteroids, matrix metalloproteinase inhibitors, and antiresorptive agents have not achieved meaningful or durable improvements in focal cartilage conditions [79,80,81]. In clinical practice, pharmacological agents should remain subordinate to rehabilitation, serving only to ease the progression of exercise and restore proper movement patterns [9,65]. For ankle lesions accompanied by effusion or acute irritation, brief use of non-steroidal agents can help reduce discomfort. In contrast, in patellofemoral cases, analgesics may facilitate participation in early strengthening and gait retraining, provided that reliance on medication does not delay active recovery [82,83]. Novel systemic agents should be used exclusively within the context of controlled trials that include transparent efficacy and safety monitoring [84].

## 6. Intra-Articular Injectables

**Figure 2:**
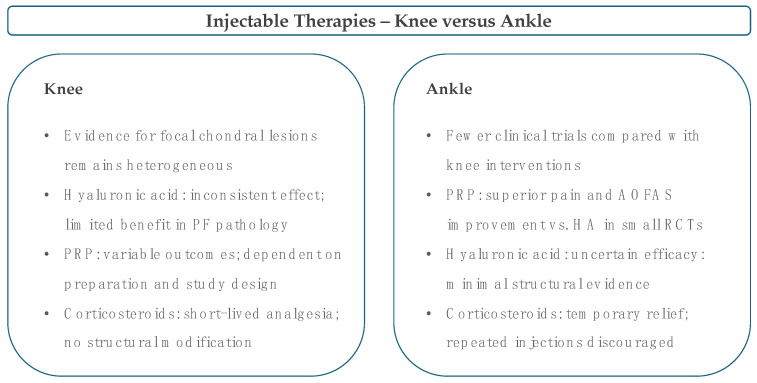
Injectable adjuncts for conservative management of focal chondral lesions in the knee and ankle.

Injectables are adjuncts to a rehabilitation-led pathway, not substitutes; indications and expectations must match joint biology and the distinct loading of the knee and ankle [4,6,17,85]. In a two-year, double-blind, randomised trial of 140 patients with symptomatic knee OA, intra-articular triamcinolone given every three months caused greater cartilage volume loss than saline (−0.21 mm vs. −0.10 mm; between-group difference −0.11 mm), without any additional improvement in pain scores [86]. These findings indicate that repeated corticosteroid injections may accelerate structural deterioration without clinical benefit and should therefore be avoided in non-arthritic focal cartilage defects, where preservation of native tissue remains the primary goal [86]. A single shot may unblock the rehab during florid synovitis. Still, it should not be framed as disease-modifying [86,87,88]. Hyaluronic acid sits in the middle; it can modulate viscosity and nociception [27,89,90,91,92]. In a randomised controlled trial involving 86 patients with patellofemoral pain who had previously failed conservative management, Hart et al. [89] compared a single 6 mL intra-articular hyaluronic acid injection with a sham procedure, with all participants following a structured home exercise programme. Both groups showed significant improvement in pain and functional scores at six months, but there was no difference between treatments in any outcome measure, including pain, KOOS, or Kujala score [89]. These findings indicate that hyaluronan provides no additional benefit over exercise alone and should therefore be reserved, if used at all, for short, clearly defined adjunctive courses within supervised rehabilitation [89]. Platelet-rich plasma offers the clearest human signal in the ankle. A randomised trial on talar osteochondral lesions, including about thirty participants, showed that three weekly leukocyte poor injections were superior to hyaluronan at twenty-eight weeks for AOFAS and pain with minimal adverse events. A pragmatic approach is two or three injections of leukocyte poor PRP after a high-quality programme has plateaued. The knee focal literature is thinner and often contaminated by OA, so consider PRP only in carefully selected non-arthritic phenotypes, with expectations set on symptomatic and functional gains rather than structure [93,94]. Bone marrow aspirate concentrate and mesenchymal stromal cell injectables remain investigational in purely non-operative focal care; most human series are used as surgical adjuncts or in OA cohorts. No randomised evidence supports the efficacy of injection only in knee or ankle focal defects. These products should be tested in trials with transparent cell characterisation and safety monitoring; regeneration claims should be made frankly [95,96,97,98,99,100,101]. Cell-free biologics, such as exosomes and stand-alone hydrogels, lack clinical evidence for focal lesions and are not routinely used; timing is crucial. Any injection should open a window to progress load and skill within two to six weeks; otherwise, palliation is mistaken for recovery, a common and unhelpful progression. A practical overview of injectable options for the knee and ankle is provided in Figure 2.

In the knee, evidence for focal chondral lesions remains heterogeneous; hyaluronic acid shows inconsistent benefit, especially in patellofemoral pathology, platelet-rich plasma (PRP) provides variable outcomes depending on preparation and study design, and corticosteroids offer only short-lived analgesia without structural modification. For the ankle, fewer clinical trials are available. Still, small randomised controlled studies suggest superior pain relief and AOFAS improvement with leukocyte-poor PRP compared with hyaluronic acid. In contrast, the efficacy of hyaluronic acid remains uncertain, and repeated corticosteroid injections are discouraged.

## 7. Nutraceuticals and Adjuncts

Nutraceuticals should be viewed as supportive measures aimed at alleviating symptoms or correcting biological imbalances, with rehabilitation remaining the central component of treatment. Evidence in humans for focal chondral defects of the knee or ankle is limited, so expectations should be cautious [102,103]. Vitamin D deficiency is common, and testing with targeted repletion is reasonable; however, supplementation has not demonstrated benefits for pain or cartilage health, discouraging high-dose use in the absence of confirmed deficiency [87,88,104]. Evidence supporting the use of glucosamine and chondroitin in focal lesions is limited and inconsistent [105]. Collagen hydrolysates have shown modest reductions in exercise-related pain in active individuals, but, without imaging evidence of cartilage repair, they are considered only short-term symptomatic adjuncts [106,107]. Undenatured type II collagen has demonstrated minor symptomatic benefits in small trials, but its role in young, non-arthritic patients remains unproven [108,109]. Other supplements, such as omega-3 fatty acids, curcumin, resveratrol, avocado-soybean unsaponifiables, and methylsulfonylmethane, possess weak or indirect evidence and cannot be recommended for cartilage regeneration [110]. Oral hyaluronan and mixed “joint complex” products lack supportive data [111]. For competitive athletes, supplement quality control and anti-doping compliance must be ensured [112]. Overall, vitamin D supplementation is advised only when deficient; glucosamine and chondroitin are not routinely recommended; and collagen peptides may be considered for targeted short-term symptom relief, provided all nutraceuticals remain secondary to rehabilitation, load management, and evidence-based injectable treatments [113]. A concise overview of the expected clinical outcomes of the currently available conservative treatment options for focal chondral lesions of the knee and ankle is summarised in Table 2.

## 8. Knee Versus Ankle: Presentation, Pathophysiology and Biomechanics

Knee and ankle chondral defects share nociception and synovial irritability yet live in distinct mechanical theatres that dictate symptoms, dosing and timelines. In the knee, the patellofemoral compartment adds substantial shear with pain on stairs, squatting and prolonged sitting. Tibiofemoral lesions more often cause load-related ache and episodic effusion during cutting or pivoting. Maltracking and dynamic valgus magnify lateral facet pressure and interact with quadriceps weakness and delayed gluteal activation, so relief depends on restoring proximal control, improving sagittal loading and refining patellar mechanics. In the ankle, the talar dome is highly congruent and compressive, with contact areas that are small and peak pressures that are high Patients report deep ache in stance and push off, with marrow oedema or cysts perpetuating pain even when the surface appears intact; hence, early deloading with protected range and rapid transition to proprioception and peroneal retraining precede impact [100,114]. Evidence quantifies these choices. In patellofemoral chondromalacia, a three-week semi squat block outperformed straight leg raises for quadriceps strength and pain, supporting closed-chain preference and short, frequent, quality-controlled sessions. Arthroscopy added to exercise did not improve nine-month outcomes in anterior knee pain, reinforcing a conservative first pathway. A medially directed realignment brace added to supervised therapy improved pain and function at six and twelve weeks with attenuation by one year, positioning bracing as a time-limited accelerator not a substitute. Immobilising stabilisers caused early quadriceps atrophy and less range without clear protection, therefore they are avoided in chondral phenotypes. In the ankle, return to hopping and direction change is gated by pain-free symmetry and acceptable time to stabilise rather than calendar time [55,56,60]. Injectables must respect joint mechanics, a single six-millilitre hyaluronan injection did not beat sham at six months in patellofemoral pain when all patients trained, so any trial should be a short series alongside supervised therapy with explicit stop rules. In talar osteochondral lesions, a randomised trial with about thirty participants found three weekly leukocyte poor platelet-rich plasma injections superior to hyaluronan at twenty-eight weeks for pain and AOFAS with minimal adverse events, which places platelet-rich plasma as the clearest ankle specific adjunct once high-quality rehabilitation has plateaued. Corticosteroid remains a brief rescue only, repeated courses are avoided, expected kinetics differ, knees with marked movement faults need longer for motor re training, and ankles may improve faster yet relapse if impact outruns proprioception, so do not let injections carry the load and use any symptomatic window to progress training, otherwise short palliation is mistaken for recovery [89,93].

Clinical progression during conservative treatment should follow a structured and transparent pathway. An initial rehabilitation phase of six to twelve weeks remains the cornerstone, focusing on pain control, movement quality, and gradual load exposure [115]. Progress must be measured using validated outcome tools, such as KOOS, IKDC, or Kujala for the knee, and FAAM for the ankle, alongside practical performance tests, including single-leg squat, hop symmetry, and timed stance [8]. A meaningful reduction in pain and restoration of function within this window indicates that the current programme can safely continue [8]. In contrast, recurrent effusion, persistent mechanical catching, or a plateau in strength or motion should prompt reassessment [116]. For the knee, failure to restore quadriceps and hip strength symmetry above 80% or ongoing swelling after stable training loads are typical warning signs warranting renewed imaging [116]. For the ankle, repeated effusions or deep aching pain during stance or push-off despite good proprioceptive recovery should raise similar concern [117]. When symptoms persist beyond twelve weeks despite adherence and correct dosing, a follow-up magnetic resonance scan helps identify subchondral changes, cystic evolution, or unstable flaps that may not be clinically visible [117]. Immediate re-evaluation is mandatory if the joint develops acute locking, gross instability, or rapidly increasing effusion [118,119].

## 9. Indications, Patient Selection and Monitoring

Conservative care is appropriate for symptomatic focal chondral or osteochondral lesions in otherwise non-arthritic knees and ankles when patients commit to structured rehabilitation. Imaging should reveal a stable defect without an unstable flap, gross malalignment, advanced subchondral collapse, or significant meniscal or ligamentous insufficiency that would impair load sharing. A pragmatic knee profile involves an isolated femorotibial or patellofemoral lesion with an intact or minimally compromised subchondral plate, occupying a small to moderate area with preserved margins and no full-thickness undercutting. The alignment should be neutral or near-neutral, and pain must be load-related rather than constant. For the ankle, ideal candidates have non-displaced talar dome lesions, limited marrow oedema, no cystic deepening affecting the bony bed, and no mechanical locking. Additionally, there should be a clinically testable pathway to restore proprioception and peroneal control [17,100,120]. The baseline assessment should include patient-reported outcomes, performance tests, and an MRI. Record KOOS or IKDC and, when patellofemoral, Kujala. For the ankle, use FAAM activities and sport; capture pain on a numerical scale at rest and during a standard task. For the knee, assess stairs; for the ankle, perform single-leg hops. Document single-leg squat quality using frontal plane projection angle, step-down control, timed single-leg stance, hop symmetry, and landing time to stabilise. Include heel-rise endurance for the ankle and simple strength metrics, such as isometric or handheld dynamometry, for the quadriceps and hip abductors. MRI confirms stability, maps marrow oedema, and inspects surrounding cartilage and plate; quantitative sequences are optional for research [121]. The Magnetic Resonance Observation of Cartilage Repair Tissue (MOCART) score (Table 3) can be used to monitor the development of the chondral defect [122]. However, its clinical validity is limited and has not been validated [123]. Follow-up imaging is not routine and is reserved for plateau, persistent effusion, or pre-procedure planning [124,125].

Progression depends on time and goals, aiming for meaningful clinical improvement within six to twelve weeks. Improvement is assessed through PROMs (considering the minimum clinically important difference, MCID, see Table 4) and objective signs such as reduced need for dynamic valgus during step downs or achieving pain-free, symmetric hops. If goals are met, increase load and skill levels following a written plan for returning to running and changing direction. If progress stalls despite fidelity and dose checks, consider an adjunct injection, such as platelet-rich plasma, in a non-arthritic talar lesion to facilitate training. Escalate care or seek surgical opinion if there is true locking, recurrent gross instability, repeated effusions limiting progression, failure to reach milestones by twelve weeks, or structural issues such as large unstable flaps, cystic deepening, or uncompensated malalignment. Monitor progress at baseline, six, and twelve weeks, then every three to six months. Ensure decisions are shared, pragmatic, and honest, with no progression by inertia [17,126].

## 10. Evidence Gaps and Research Priorities

Non-operative care for focal chondral lesions suffers from inconsistent definitions, heterogeneous dosing of rehabilitation and injectables, and short follow-up, which together obscure true effect size and durability. Priorities begin with standardisation. Trials should adopt shared eligibility thresholds that separate focal non arthritic defects from OA, stratify by lesion size, depth, stability and location, document alignment, instability and meniscal status, and report a reproducible rehabilitation backbone with quantified exposure, for example, sets, frequency, external load, foot contact strategy and graded impact progression, as well as any bracing or taping dose and weaning rules. Hyperbaric oxygen therapy has biological plausibility in focal cartilage injury because increased tissue oxygen tension can dampen inflammatory signalling, support mitochondrial function and influence chondrocyte survival and matrix synthesis [127,128]. Human evidence specific to focal chondral defects of the knee or the ankle is, however, very limited. No randomised trial has tested hyperbaric oxygen against sham or standard care in patients with imaging-confirmed focal defects. There are no established recommendations on the use of hyperbaric oxygen in clinical practice, and it should therefore not be included in a routine conservative algorithm for focal chondral lesions of the knee or the ankle. Injectables require harmonised preparation and timing, platelet-rich plasma trials should report platelet dose, leukocyte content, activation, number and spacing of injections, and their relation to exercise sessions, hyaluronan studies should specify molecular weight and series length, and any cell-based protocol should include cell source, characterisation, viability and release criteria, with cell-free biologics confined to early phase safety work. Factorial non-operative designs are needed because benefit likely arises from combinations rather than isolated inputs. Pragmatic multicentre trials that randomise patients to rehabilitation with or without a brace and with platelet-rich plasma, sham, or hyaluronan can disentangle interaction effects and yield clinically actionable answers. Crossover or adaptive features may increase efficiency while preserving internal validity. Outcome measurement should blend symptoms, function, performance and structure, pain and function can be tracked with joint appropriate instruments such as KOOS or IKDC for the knee and FAAM for the ankle, plus a compartment-specific score where relevant. Predefined minimal important change thresholds should be declared in the protocol, performance should include single-leg squat quality, step down control, hop testing and time to stabilise, with return to running and change in direction gated by objective criteria rather than calendar time. Structural endpoints should be incorporated without overpromising. Quantitative MRI, such as T2 or T1 rho mapping and modern morphological scores, can document tissue status, while ankle studies should add computed tomography or high-resolution MRI to assess subchondral cysts and plate integrity. Imaging should be scheduled to answer mechanistic questions rather than performed reflexively. Durability and safety require long horizons, at least two years for knee and ankle cohorts, with standardised adverse event definitions that include post-injection flares, effusions limiting training, infections, and unplanned procedures, as well as capture of sport exposure and workload to contextualise outcomes. Finally, registries with a lean core dataset should be established for focal knee and talar lesions, collecting lesion mapping, rehabilitation dose, adjuncts, patient-reported outcomes and key performance tests at fixed intervals, and using a joint specific core outcome set agreed by clinicians, patients and researchers. This infrastructure will support external validity, facilitate benchmarking across centres and help identify phenotypes that truly benefit from conservative strategies.

## 11. Future Prospects

The management of focal chondral lesions is expected to become increasingly individualised, with treatment tailored to the biological and mechanical characteristics of each joint. Advances in imaging, motion analysis, and biomarker profiling now enable a more comprehensive understanding of tissue quality, adaptive capacity, and lesion environment. Future strategies are likely to define patient phenotypes based on cartilage integrity, subchondral bone condition, inflammatory tone, neuromuscular control, and loading patterns, rather than solely on lesion size. This will support a more precise selection of conservative interventions, from targeted rehabilitation protocols to specific injectable combinations, adapted to each context. Artificial intelligence may further refine this process by identifying predictive patterns in clinical and imaging datasets, while wearable sensors will enhance monitoring and adherence. Alongside these technological advances, an integrative, multimodal concept of pain management is expected to gain prominence, combining physical reconditioning, pharmacological support, and psychological components to optimise recovery. This comprehensive model recognises that pain and function are influenced not only by local pathology but also by behavioural and central factors. Ultimately, a phenotype-driven, data-informed, and multimodal approach that merges biological insight with mechanical precision will likely define the next phase of conservative management for focal cartilage lesions, enabling durable outcomes and reducing the need for surgical intervention.

## 12. Conclusions

Rehabilitation remains the central element of conservative management for focal chondral lesions of the knee and ankle. Structured, progressive exercise guided by clear functional criteria provides the most consistent evidence for pain reduction and functional recovery. Adjunct treatments, including intra-articular injectables or selected nutraceuticals, should be viewed only as supportive tools that facilitate rehabilitation rather than replace it. Symptom relief following an injection must never be interpreted as evidence of structural repair; instead, any temporary improvement should be used to advance strength, coordination, and load tolerance within a supervised training plan. Durable outcomes depend on disciplined, criterion-based progression, objectively documented through simple, repeatable measures such as strength symmetry, hop performance, and validated patient-reported outcomes. Conservative care, therefore, succeeds when it remains active, measurable, and biologically and mechanically coherent, allowing improvement in function and quality of life without the false reassurance of transient symptom relief.

## Figures and Tables

**Table 1:** Strings used for the search in each database (WoS: Web of Science).

PubMed	Scopus	WoS
(“Cartilage, Articular”[MeSH] OR “chondral defect” OR “chondral de-fects” OR “cartilage lesion” OR “osteochondral lesion” OR “focal carti-lage defect” OR “cartilage injury” OR “cartilage repair”) AND (“treat-ment “[MeSH] “outcome”[MeSH] “management”[MeSH] “conserva-tive”[MeSH] OR outcome OR efficacy OR safety OR “magnetic reso-nance imaging” OR MRI OR histology OR “clinical evaluation” OR “functional outcome” OR “patient reported outcome” OR PROM OR complication OR failure OR “return to sport”)	TITLE-ABS-KEY(“chondral defect” OR “chondral defects” OR “cartilage lesion” OR “osteochondral lesion” OR “cartilage injury” OR “cartilage repair”) AND TITLE-ABS-KEY(“outcome” OR treatment OR efficacy OR safety OR MRI OR histology OR “clinical evaluation” OR “functional out-come” OR “patient reported outcome” OR PROM OR complication OR failure OR “return to sport”)	TS = (“chondral defect” OR “chondral defects” OR “cartilage lesion” OR “osteochondral lesion” OR “cartilage injury” OR “cartilage repair”) AND TS = (“treatment” OR outcome OR efficacy OR safety OR MRI OR histology OR “clinical evaluation” OR “functional outcome” OR “patient reported outcome” OR PROM OR complication OR failure OR “return to sport”)

**Table 2:** Summary of outcomes of current conservative treatments for focal chondral lesions of the knee and ankle (OA: osteoarthritis; PRP: platelet-rich plasma; AOFAS: American Orthopaedic Foot & Ankle Society score; NSAIDs: non-steroidal anti-inflammatory drugs).

Treatment	Main Clinical Outcome in Focal Knee/Ankle Lesions	Key Limitations/ Structural Effects
**Structured rehabilitation and load management**	Consistent short- to mid-term improvements in pain, function and return to sport when delivered as a 6–12-week, criterion-based programme focusing on closed-chain strengthening, neuromuscular control and education.	Cornerstone of care but not reliably associated with structural cartilage restoration; success depends on adherence, high-quality supervision and correct dosing.
**Oral pharmacotherapy**	Short courses reduce pain and synovitis sufficiently to enable full participation in rehabilitation, particularly during symptomatic flares.	Supportive rather than disease-modifying; no evidence of cartilage repair and long-term use is limited by gastrointestinal, renal and cardiovascular adverse events.
**Bracing and taping**	Medially directed patellofemoral braces and taping can improve pain and function in the short term and facilitate exposure to strengthening and gait retraining.	Effects attenuate over time; motion-restricting stabilising braces may cause quadriceps atrophy and reduced range of motion and are therefore discouraged in chondral phenotypes.
**Intra-articular corticosteroids**	Single injections may provide short-lived analgesia and reduce florid synovitis, occasionally unblocking rehabilitation.	Repeated courses in knee OA accelerate cartilage volume loss without sustained clinical benefit; thus, not recommended as a disease-modifying or repetitive treatment for non-arthritic focal defects.
**Intra-articular hyaluronic acid (HA)**	In patellofemoral pain, a single 6 mL injection combined with exercise improved symptoms, but not more than sham plus exercise; ankle data remain limited and heterogeneous.	Acts mainly as a symptomatic adjunct with no proven structural repair; should be reserved, if used at all, for short, clearly defined adjunctive courses within supervised rehabilitation.
**Platelet-rich plasma (PRP)**	In a randomised trial of talar osteochondral lesions, leukocyte-poor PRP provided superior pain relief and AOFAS improvement at 28 weeks compared with hyaluronan, suggesting meaningful short- to mid-term clinical benefit after high-quality rehabilitation has plateaued.	Evidence for focal knee lesions is thinner and often confounded by osteoarthritis; structural regeneration is unproven and expectations should focus on symptomatic and functional gains rather than cartilage repair.
**Nutraceuticals**	May offer modest symptomatic relief in selected patients (e.g., collagen peptides in activity-related pain), but data in focal knee and ankle chondral defects are limited.	No convincing imaging evidence of cartilage regeneration; vitamin D is recommended only in deficiency, glucosamine/chondroitin are not routinely advised, and all nutraceuticals should remain secondary to rehabilitation and evidence-based injectables.

**Table 3:** The MOCART score.

Volume Fill of Cartilage Defect	
Complete filling OR minor hypertrophy: 100% to 150% filling of total defect volume	20
Major hypertrophy ≥150% OR 75% to 99% filling of total defect volume	15
50% to 74% filling of total defect volume	10
25% to 49% filling of total defect volume	5
<25% filling of total defect volume OR complete delamination in situ	0
**Integration into adjacent cartilage**	
Complete integration	15
Split-like defect at repair tissue and native cartilage interface ≥ 2 mm	10
Defect at repair tissue and native cartilage interface > 2 mm, but < 0% of repair tissue length	5
Defect at repair tissue and native cartilage interface ≥ 50% of repair tissue length	0
**Surface of the repair tissue**	
Surface intact	10
Surface irregular < 50% of repair tissue diameter	5
Surface irregular ≥ 50% of repair tissue diameter	0
**Structure of the repair tissue**	
Homogeneous	10
Inhomogeneous	0
**Signal intensity of the repair tissue**	
Normal	15
Minor abnormal—minor hyperintense OR minor hypointense	10
Severely abnormal—almost fluid-like OR close to subchondral plate signal	0
**Bony defect or bony overgrowth**	
No bony defect or bony overgrowth	10
Bony defect: depth < thickness of adjacent cartilage OR overgrowth < 50% of adjacent cartilage	5
Bony defect: depth ≥ thickness of adjacent cartilage OR overgrowth ≥ 50% of adjacent cartilage	0
**Subchondral changes**	
No major subchondral changes	20
Minor oedema-like marrow signal—maximum diameter < 50% of repair tissue diameter	15
Severe oedema-like marrow signal—maximum diameter ≥ 50% of repair tissue diameter	10
Subchondral cyst ≥5 mm in longest diameter OR osteonecrosis-like signal	0
**Total score (0–100)**	

**Table 4:** MCIDs of the most commonly used PROMs (IKDC = International Knee Documentation Committee; KOOS = Knee injury and Osteoarthritis Outcome Score; ADL = Activities of Daily Living; QoL = Quality of Life; WOMAC = Western Ontario and McMaster Universities Osteoarthritis Index; SF-36 = Short Form Health Survey 36; MCS = Mental Component Summary; PCS = Physical Component Summary; CKRS = Cincinnati Knee Rating Scale).

PROM (Score Range)	MCID
IKDC (0–100)	17.4
KOOS–ADL (0–100)	10
KOOS–Pain (0–100)	13.4
KOOS–QoL (0–100)	13.4
KOOS–Sports/Rec (0–100)	19.2
KOOS–Symptoms (0–100)	19.2
WOMAC–Pain (0–20)	7.5
WOMAC–Physical Function (0–68)	5.9
WOMAC–Stiffness (0–8)	18.8
WOMAC–Overall (0–96)	11.5
Tegner Lysholm (0–100)	19.2
SF-36–MCS (0–100)	0.3
SF-36–PCS (0–100)	4.6
SF-36–Physical Functioning (0–100)	17.5
SF-36–Role Physical (0–100)	12.5
SF-36–Vitality (0–100)	2.6
CKRS (6–100)	26

## Data Availability

The datasets generated during and/or analysed during the current study are available throughout the manuscript. No new data were created or analysed in this study.
